# Application and evaluation of transitory protective stoma in ovarian cancer surgery

**DOI:** 10.3389/fonc.2023.1118028

**Published:** 2023-03-24

**Authors:** Jiaqi He, Jinke Li, Bao Fan, Liya Yan, Ling Ouyang

**Affiliations:** Department of Obstetrics and Gynecology, Shengjing Hospital of China Medical University, Shenyang, China

**Keywords:** transitory protective stoma, ovarian cancer, cytoreductive surgery, gynecological oncology surgery, colorectal resection

## Abstract

Ovarian cancer is the most fatal of all female reproductive cancers. The fatality rate of OC is the highest among gynecological malignant tumors, and cytoreductive surgery is a common surgical procedure for patients with advanced ovarian cancer. To achieve satisfactory tumor reduction, intraoperative bowel surgery is often involved. Intestinal anastomosis is the traditional way to restore intestinal continuity, but the higher rate of postoperative complications still cannot be ignored. Transitory protective stoma can reduce the severity of postoperative complications and traumatic stress reaction and provide the opportunity for conservative treatment. But there are also many problems, such as stoma-related complications and the impact on social psychology. Therefore, it is essential to select appropriate patients according to the indications for the transitory protective stoma, and a customized postoperative care plan is needed specifically for the stoma population.

## 1 Introduction

Ovarian cancer (OC) is the most fatal of all female reproductive cancers (1). The fatality rate of OC is the highest among gynecological malignant tumors. Its main characteristics are insidious onset, high malignancy and strong invasive ability. And most patients are diagnosed only in the middle and late stages of development. Cytoreductive surgery is a common surgical procedure for patients with stage III and above. Residual disease after primary surgery for ovarian cancer is the strongest prognostic factor affecting patient survival (2). More precisely, a 10% increase in the likelihood of achieving maximal or optimal cytoreductive surgery (no gross residual disease) is associated with a 5.5% increase in the average survival rate (3). The metastasis of advanced ovarian cancer is mainly by direct spread and intra-pelvic and abdominal dissemination, which can invade all segments of the intestinal canal. The rectosigmoid colon is anatomically close to the female pelvic organs and is the most commonly involved part of the intestine, accounting for 24-64% (4), followed by the ileocecal colon (5). Therefore, optimal tumor cytoreduction in advanced primary and recurrent disease usually requires radical upper gastrointestinal surgery and bowel segment resection (6). Total mesorectal resection (TME) is a commonly used surgical technique in colorectal surgery (7). It refers to the ligation of the superior rectal artery, through the areolar avascular plane along the mesorectum fascia down to the pelvic floor. Its advantage is that it can reduce the local recurrence rate (8), but increase the risk of local complications (9, 10). However, ovarian cancer cells primarily disseminate within the peritoneal cavity and are superficially invasive in most cases. In specific cases where no lymph node lesions are found at the beginning of the inferior mesenteric artery and the ratio of the length of the left colon to the extent of the colorectal cancer lesions is good, mesorectal-sparing resection with preservation of the superior rectal artery and mesorectal tissue can be performed, thus preserving the rectal mesenteric tissue and vascular supply (11). This method not only reduces the probability of local complications, but also ensures satisfactory tumor reduction. After intestinal surgery, there are usually some intestinal-specific complications. Anastomotic leakage (AL) is one of the most serious complications. In the OC study, the main risk factors were advanced age, low serum albumin level, multiple small bowel resection, manual anastomosis and short distance from anastomosis to anal margin (12). In colorectal surgery, surgeons usually use visual assessment and air leakage test to assess anastomotic rectal perfusion. Recently, gynecologists have found that near-infrared (NIR) angiography *via* proctoscopy can be used to evaluate rectal perfusion after rectosigmoidectomy and anastomosis. It provides an opportunity to identify poor perfusion, leading to the reinforcement or revision of the anastomosis (13). Transitory protective stoma (TPS) is another commonly used procedure to reduce the incidence of AL. It refers to pulling the small intestine out of the abdominal cavity and making a stoma in the abdominal wall after colorectal resection and anastomosis. It reduces complications by leaving out the intestines at the site of colorectal anastomosis. This article will describe the development for advanced ovarian cancer with digestive tract metastasis and the application status and value of TPS in ovarian cancer surgery.

## 2 Development of treatment for advanced ovarian cancer with digestive tract metastasis

Initially, gynecologic oncologists often perform end-to-end anastomosis after modified posterior pelvic exenteration (MPE) or colorectal resection to restore continuity of the gastrointestinal tract to achieve optimal cytoreduction (14, 15). An ideal anastomosis requires an adequate blood supply, tension-free serosal apposition, and an uncompromised lumen with a watertight seal (16). The two common methods of constructing an anastomosis are handsewn anastomosis (HA) and stapled anastomosis (SA). HA has been used for more than a century, and over time, various HA techniques have been invented and developed, including single-layer versus double-layer and inverting versus everting sutures. Historically, double-layer sutures were the technique of choice until the late 1970s. However, the double-layer suture is more technically challenging because it requires identifying each layer of the bowel and then separating them appropriately, which makes it more difficult to maintain suture tension. The higher demands on technique result in an increased risk of failure with double-layer sutures. Because it takes longer than single-layer sutures, excessive tissue handling can lead to severe tissue damage and avascular necrosis of the tendon sleeve, which can lead to anastomotic rupture. As an alternative, single-layer sutures became popular in the early 1980s. Single-layer sutures reduce the potential risk previously explained. But several authors have suggested that single-layer sutures also increase the risk of dehiscence because the suturing technique uses a submucosal suture technique with the serosal layer (17). For the use of inverting versus everting sutures, since the publication of Lembert, surgeons have generally advocated inverting sutures because it was thought that mucosal protrusion would lead to anastomotic rupture. However, in the 1960s, two clinical studies (18, 19) showed that good healing could also be achieved with an everting suture. In a series of subsequent studies, there was still no consensus conclusion (20–26). However, regardless of the type of manual suture, it causes a series of inflammatory reactions because dragging the suture through the intestinal wall could damage the tissue (23). Compared to non-absorbable or slowly absorbable sutures, absorbable sutures cause more tissue reactions (27–29), while compromising the strength of the anastomosis by dissolving too quickly. Therefore, non-absorbable sutures may be a better choice.

SA was recommended after validation in relatively small randomized trials conducted between 1970 and 2009 (30). The instrument is similar to a sigmoidoscope with a handle, except that the obturator (nose cone) of the sigmoidoscope protrudes beyond the cylinder. For a low rectal anastomosis, the instrument is inserted through the anus with the nose cone protruding distally. The distal end of the rectum is secured to the staple-carrying cylinder of the instrument with a purse-string suture. And the proximal rectum tied in a purse-string over the anvil-carrying nose cone. The nose cone is then aligned with the cylinder and the two ends of the bowel are anastomosed after excitation of the instrument (31). SA ensures uniformity of the suture and reduces tissue manipulation, so the inflammatory irritation caused is very mild and facilitates the healing of the incision. It also has a shorter operating time and hospital stay (32). The technical requirements are lower than for HA.

Although bowel resection-anastomosis is safe and feasible in PDS, its possible complications still cannot be ignored. AL is a common and serious complication after intestinal anastomosis (33). It has an incidence of 1.26%-9% in patients with ovarian cancer (34). There is no standard definition of AL, and most studies define it as unclean fluid draining from the stoma, incision, or vagina, definitive imaging evidence of anastomotic leakage, or AL confirmed by reoperation (33, 35). AL has now been shown to be an independent risk factor for reduced overall patient survival after cytoreductive surgery for ovarian cancer. Its development has been associated with multiple factors in the perioperative period. Advanced age, multiple bowel resections, low serum albumin levels (≤3 g/dL), and short distance from the anastomosis to the anal verge (<5-6 cm) are independent factors associated with the formation of AL (36–38). Poor postoperative blood supply to the intestinal anastomosis, hypertonicity, and a sharp increase in intestinal pressure during defecation and exhaust are also common causes of AL (39). This surgical complication leads to adverse oncological outcomes due to higher postoperative mortality and delayed duration of adjuvant chemotherapy (33). Adjuvant chemotherapy cannot be initiated in approximately one-third of AL patients (35).

In the field of colorectal surgery, the incidence of AL in the two surgical plans for constructing anastomosis was vary between studies published by different research centers. After analyzing studies of 1125 patients in seven randomized controlled trials published before 2009, Choy et al. found significantly fewer anastomotic leaks in SA compared to HA during ileocolic resection (30). Luglio G et al. updated the systematic review by Choy et al. in 2019 to include 6 trials and 995 patients (825 with cancer), in which SA had significantly less anastomotic leak than HA (1.3% vs. 6.6%; OR 0.28) (40), consistent with previous findings. But in a recent large sample analysis by Nordholm et al. the incidence of AL was found to be two times higher after surgery with SA than with HA, contrary to the results of a previous meta-analysis (41) In their study, Espin noted that the effect of AL on HA patients was much less than that on SA patients, and that HA patients were much less likely to have AL followed by reoperation after surgery and could mostly be cured by conservative treatment (42). Analyzing the results of studies from different centers, it is easy to see that HA is more challenging for the operator and takes longer to perform; SA is suitable for less experienced operators, but is more expensive (43). In low-risk procedures such as elective or laparoscopic surgery, the operator prefers to apply anastomosis, while in high-risk procedures such as emergency surgery and laparotomy, HA is more beneficial to reduce the incidence of AL, but also requires a more qualified operator to operate (44). A recently published study by Enomoto H et al., 2022 evaluated the incidence of anastomosis-related complications associated with the establishment of anastomosis after transanal total mesorectal excision for low rectal cancer using the stapler plus reinforced sutures procedure. All patients using the SA underwent additional HA on each round of the anastomotic line. The incidence of anastomosis-related complications, pelvic abscesses, and anastomotic stenosis was found to be significantly lower in this group of patients compared with HA (P < 0.001, P < 0.048, and P < 0.032) (45). This study provides a new idea for anastomotic construction, combining both anastomotic methods to achieve a reduction in associated complications.

In the field of gynecologic oncology, bowel surgery has become very common in PDS of advanced ovarian cancer. It benefited from the extensive surgical experience in colorectal surgery. But the higher rate of complications, especially AL, has not been fundamentally solved. When serious complications such as AL occur after intestinal anastomosis, they can only be addressed by the use of broad-spectrum antibiotics, placement of pelvic drainage, interventional radiotherapy, or secondary surgery (46, 47). Therefore, gynecologic oncologists urgently need a better approach to reduce the incidence of complications after intestinal anastomosis and to achieve a better prognosis for their patients.

## 3 Transitory protective stoma in PDS

### 3.1 Necessity and indications for performing a transitory protective stoma

The current best treatment option for advanced ovarian cancer is a combination of surgery to reach R_0_ and postoperative treatment with first-line chemotherapy. After colorectal resection during primary cytoreductive surgery, TPS has been introduced by gynecologic oncologists for the surgical treatment of advanced ovarian cancer in order to protect the distal colorectal anastomosis and facilitate the healing process. TPS is one of the more commonly used procedures in colorectal surgery. It involves the placement of a drainage tube in the pelvis after the colorectum has been resected and anastomosed. And before the abdominal wall is closed, a small circular piece of skin is incised at a previously marked point, the underlying fatty tissue is removed, and the fascia is opened to accommodate three fingers. A small segment of ileum (usually located 10-15 cm proximal to the ileocecal) is pulled out of the abdominal cavity through the skin incision and the intestinal tube is incised and gently turned outward to create the stoma, which is looped and secured to the skin, and finally the stoma bag is adhered to the skin. By this method, the colorectal anastomosis is kept vacant to avoid the passage and contamination of feces. If end-to-end anastomosis is performed after gastrointestinal resection in traditional, a seven-day fast is usually required to reduce complications such as AL and peritoneal sepsis. Parenteral nutrition exacerbates postoperative stress in patients and may also affect the timing of the first postoperative chemotherapy. Compared with HA or SA, leaving a TPS for shunting allows patients to resume eating as soon as possible after surgery, ensures enteral nutrition supply, reduces postoperative stress in patients, and starts adjuvant chemotherapy on time. More importantly, it can reduce the incidence of AL (48–50), reducing the likelihood of emergency reoperation and transfer to ICU treatment after AL. Retrospective studies have reported the incidence of AL in TPS patients ranging from 4.9% to 8.2%, compared with 16%-17% in non-TPS patients (51, 52). A Meta-analysis by Montedori and Huser showed that TPS reduced the incidence of clinical AL (ORs of 0.33, 0.39, and 0.32, respectively) and reoperation rates (ORs of 0.23, 0.29, and 0.27, respectively) (53–55). TPS is particularly important in patients with anastomotic stenosis, immunosuppression, or active infection, and its use protects the newly created intestinal anastomosis from becoming the culprit of pelvic sepsis or systemic disease (56). Although other studies have also concluded that it does not reduce the incidence of AL, it may still reduce the incidence of complications such as sepsis or reopening surgery. Even when AL occurs, the incidence of perianastomotic inflammation, pelvic infection, or diffuse peritonitis is significantly lower and less severe, and can usually be cured by conservative treatment (57).

In ovarian cancer surgery, several studies have demonstrated the tolerability of TPS in patients (37). However, there are few reports on the use of TPS in PDS for ovarian cancer and patient selection is challenging, mainly due to the lack of established preoperative and intraoperative predictors of AL in this population. In the colorectal cancer literature, Maurizio et al. constructed a digital nomogram by collecting large samples of Canadian patients, including body mass index, sex (male), tumor location (middle and low vs. high), type of approach (MIS vs open), number of cartridges employed (1vs.>1), weight loss, clinical T and combined multiple organ resection and other covariables significantly associated with postoperative AL. Based on this nomogram, a specific risk score (RALAR score) is constructed to calculate the probability of AL, which can help surgeons make decisions about recommending TPS (58). In OC surgery, there is a temporary lack of similar predictive models, and most of the use of TPS is based on the conclusions of retrospective analysis or the surgical experience of gynecological oncology surgeons. After a multidisciplinary meeting with colorectal surgery specialists in their clinic, Kalogera et al. developed a protocol by comparing optimal timing and reviewing relevant literature to determine specific criteria for implementing a TPS. They concluded that a TPS was recommended if any of the following criteria were met: preoperative albumin ≤3.0 g/dL; previous pelvic radiation therapy; colectomy; anastomosis ≤6 cm from the anal verge; signs of bowel compromise; intra-operative leak on air-leak test; gross contamination or surgeon’s concern. Of the 77 patients evaluated, 27 (35%) underwent a TPS. The AL rate for this study was 1.3%, compared with a historical AL rate of 7.8% (37). In contrast, in the study by Jill H. Tseng et al, only two factors were significantly associated with the decision to perform TPS on patients in PDS: longer operative time and longer length of rectosigmoid resection. They highlighted that the individual attending surgeon was not associated with the formation of TPS in univariate or multivariate analyses. It differs from the study by Kalogera et al. They suggest that this reflects the views of a large group of gynecologic oncology surgeons - longer operative time represents higher surgical complexity and longer length of rectosigmoid resection represents higher anastomotic tension. In that study cohort, the AL rate for patients with stomas was 4.5% (57). We also searched the Pubmed database for other relevant literature listing the causes of TPS formation in cytoreductive surgery for ovarian cancer (**Table 1**). A total of 2868 patients were included in 12 articles, with stoma rates ranging from 1.7% to 58%. Among the most likely reasons for the operator to choose TPS were a longer operative time and a lower-positioned anastomosis.

**Table 1:** Selected studies assessing TPS during gynecologic oncology surgery.

	Study population	AL rate	Stoma rate	Causes of stoma
Lago V. et al. (59)	695patients	13.9% (with DI) 8.9% (without DI)	19.13% diverting ileostomy	Previous treatment with bevacizumab Additional bowel resection Extended operating time Intra-operative red blood transfusion
Koscielny et al. (60)	136patients	16.9%	16.18% protective loop ileostomy	Not described
Jill H. et al. (57)	331patients	4.5% (with DI) 7.0% (without DI)	13.0% loop ileostomy	Longer operative time Greater length of rectosigmoid resection
Gockley AA et al. (61)	554patients	3.6%	4.5% ostomy	Longer mean operative time A high Aletti score
Tozzi et al. (62)	163patients	5%	33.7% diverting loop ileostomy	Impaired tissue quality Multiple bowel resections Anastomosis <6 cm from the anal verge Non-tension free anastomosis Spillage at air test
Canlorbe et al. (63)	99patients	1.0% (with DI) 6.1% (without DI)	10% transitory protective stoma	Not described
Shuang Ye et al. (64)	282patients	1.0% (with DI) 4.4% (without DI)	35.5% ostomy	Not described
Bristow et al. (65)	56patients	5.4%	12.5% protective colostomy/ileostomy	Tension at the anastomotic staple line Concerns over adequate vascularization of the anastomosis Local contamination from spillage of bowel contents
Susannah et al. (66)	70patients	1.7% (without DI)	17.1% diverting ileostomy	Preoperative large bowel obstruction Tenuous anastomosis Low anastomosis Extensive intraoperative blood loss Poor bowel preparation Extensive bowel resection Presacral bleeding Long-term steroid use
Fournier et al. (67)	228patients	2.89%	58% protective ileostomy	Low anastomosis Multiple bowel resection Ascites>500ml
Richardson et al. (38)	177patients	6.8%	1.7% protective stoma	An albumin level less than 3.0 g/dL
Kalogera et al. (37)	77patients	1.3%	35.1% diverting stoma	Preoperative albumin ≤3.0g/dL Prior pelvic radiation RSR plus additional large bowel resection (LBR) Anastomosis (AS) ≤6cm from the anal verge Failed leak test or contamination of the pelvis with stool

### 3.2 Preoperative preparation for transitory protective stoma

Prior to the procedure, the patient needs to have the stoma position pre-set and a mark made on the skin surface. This is a very crucial part of the preoperative process, which determines the best position for the stoma by assessing the position of the abdomen while sitting and standing. Such preparation can help reduce numerous postoperative problems such as leakage, difficulty in putting on the stoma bag, skin irritation, pain and clothing friction (68). The importance of preoperative stoma position marking has been confirmed by numerous studies. In a study by Bass et al. 292 patients with a marked stoma, location was compared to 301 with unmarked stoma. The authors reported an overall complication rate of 32.5% and 43.5% in the marked and unmarked groups respectively (P < 0.0075). The incidence of early postoperative complications was significantly higher in the unmarked group than in the marked group (69). Similar findings to Bass were obtained by Parmar et al. Patients with a preoperative marked stoma had a significantly lower risk of developing a problem stoma (20%) than patients with an unmarked stoma (55.9%) or a stoma outside the marked location (42.9%) (P < 0.001) (70). Therefore, both the American Society of Colon and Rectal Surgeons (ASCRS) and the Wound, Ostomy and Continence Nurses Society (WOCN) recommend stoma location marking (71, 72). Ideally, stoma positioning should be performed in the outpatient clinic before the operation, which allows the patient to actively participate in a low-stress environment, thereby promoting a patient-centered approach to stoma (73). Note the physical features that may affect the stoma: abdominal bulge, abdominal folds and scars, etc. (72) The patient’s ability to see the stoma, finger dexterity, clothing style preference, level of independent mobility, and preference for stoma position should also be considered. The patient’s own involvement will allow for better selection of an appropriate position based on the patient’s dressing habits and activities (74). If the stoma position is chosen incorrectly, it may hinder postoperative recovery or lead to other complications. Once the patient is anesthetized, the skin folds or creases will be reduced or disappear because the muscles are relaxed (75), and if the stoma is found in a skin fold after surgery, it may lead to mechanical leakage. Regardless of the type of stoma, preoperative stoma marking helps to successfully maintain the seal of the stoma system. And it can significantly improve the patient’s quality of life, increases the patient’s self-confidence and independence, and reduces the incidence of postoperative complications.

### 3.3 Choice of stoma solution

Ileostomy and colostomy are two frequently mentioned options when it comes to the choice of stoma type in surgery. The human intestine processes about 8L-10L of fluid per day, with the jejunum and ileum absorbing most of the fluid. It is estimated that about 1.5L of fluid reaches the colon, of which only 100mL is excreted (76). Thus, ileostomy has a higher output compared to colostomy. A stoma with a output greater than 2000 ml/d is defined as a high-output stoma (HOS). The etiology of HOS is usually unclear, although intra-abdominal sepsis, medication, intermittent obstruction and reduced small bowel length (<200 cm) account for a proportion of patients with HOS. Imaging is helpful because it can show the length of the remaining small intestine, as well as possible signs of obstruction, diverticulum, active mucosal disease, and intestinal fistula formation. If these signs are present, treatment of incomplete intestinal obstruction or intra-abdominal sepsis can resolve the high output (77). However, for HOS of unknown cause, treatment can only be done by limiting oral hypotonic fluids, oral medication to slow bowel motility before meals (78, 79), and intravenous rehydration. If persistent HOS is not properly managed, the consequences of dehydration, electrolyte disturbance, acute kidney injury and malnutrition are likely to occur (77).

However, ileostomy is not without its unique advantages. Tilney et al. studied seven studies, including three randomized controlled trials, and found that ileostomy reduced wound infection (OR = 0.21, 95% CI: 0.07, 0.62, P = 0.004) and overall complication rates (OR = 0.22, 95% CI: 0.08, 0.59, P = 0.003) (80). Comparing the use of ileostomy with colostomy in 1529 patients, Rondelli et al. showed a higher risk of dehydration due to high output but a lower risk of prolapse (OR = 0.21) and sepsis (OR = 0.54) in patients who received an ileostomy (81).

It is worth mentioning that some nutrition experts have offered new solutions to the problem of ileostomy causing HOS. Prebiotic fiber, insoluble fiber and soluble fiber are hydrolyzed or absorbed in the gastrointestinal tract. Some studies have shown that soluble fibers such as partially hydrolyzed guar gum (PHGG) can provide a dichotomous effect as a stool normalizer. It can both soften hard stools in constipation and improves the consistency of loose/liquid stools in diarrhea (82, 83). Since the use of prebiotic fibers in reducing diarrhea is still inconclusive (84), leading to soluble PHGC fiber as a possible intervention strategy to minimize stoma evacuation. Several local cases reported from Malaysia have shown that PHGC fiber helps to reduce high stoma output in colorectal cancer patients after ileostomy (85), as well as chemotherapy-induced high ileostomy output. This is an important guideline for patients with ovarian cancer who require postoperative chemotherapy in conjunction with surgery. In a recent preliminary study in nutrition, patients treated with PHGG fiber had significantly lower stoma output and achieved higher energy and protein intakes compared to patients treated with standard therapy (SG), but with a nonsignificant reduction in the hospital stay (86).

Overall, most studies have concluded that ileostomy and colostomy are functionally equivalent. The disadvantage of ileostomy is that it may produce high output, leading to acute kidney injury and small bowel obstruction due to adhesive disease, whereas the disadvantage of colostomy is the higher incidence of parastomal hernias and prolapse (87). The smaller size of ileostomy, less odor, less susceptibility to prolapse, fewer complications of fistulae, and progressive resolution of the high output problem have made ileostomy more popular with surgeons than colostomy. A comprehensive nutritional intervention program during hospitalization and post-discharge after ileostomy is essential to increase compliance with new nutritional interventions, promote recovery, and improve clinical outcomes (88). Integration of PHGG fiber supplementation in postoperative nutritional interventions and education for ileostomy patients should be part of medical nutrition therapy.

### 3.4 Perioperative education and follow-up of patients with stoma

Patients with stomas may experience physical, psychological, and social challenges after surgery (89). Richburg et al. found that the top five difficulties experienced by this group of patients after discharge were peri-stoma skin irritation (76%), ostomy bag leakage (62%), odor (59%), decreased social activity (54%), and depression and anxiety (53%) (90). Ostomy actually prolongs the length of stay for new ostomates who are otherwise ready to be discharged but feel uncomfortable and unsure about stoma management. In the AMA survey, 29% of recently discharged new ostomates were unable to empty a pouch and 56% were unable to independently apply a new ostomy bag. Regarding this issue, Deborah Nagle’s team designed a complete program for ostomy patients, including preoperative ileostomy awareness, online standardized educational materials, direct postoperative engagement of inpatients, observation of patient ostomy management, and post-discharge tracking of in and out fluid volumes (91). Jenan Younis et al. adopted a similar method, but started earlier. In addition to information and counseling at the preoperative visit, patients received a disc on stoma care and a practice kit containing self-adhesive foam stoma and ileostomy bags to practice. Before these patients were hospitalized, they had already attempted to “empty” and replace several stoma bags. With the introduction of intensive preoperative stoma education as part of the rehabilitation program, the number of patients with delayed discharge due to stoma self-care problems could be significantly reduced (92). This suggests that perioperative education should begin early for patients who are likely to undergo stoma surgery, as it can help to reduce postoperative complications and anxiety.

According to the American Society of Colon and Rectal Surgeons, optimal care for patients undergoing stoma surgery includes preoperative, perioperative, and postoperative care by expert stoma nurses. The importance of the stoma nurse has been confirmed by many studies. A large retrospective study conducted by Bass and Pittman J et al. showed that preoperative education by stoma nurses was associated with fewer stoma-related complications (23% and 32%) and a significant reduction in postoperative skin and leakage problems (69, 93). Haudhri et al. randomly assigned 42 patients to an intensive preoperative education program prior to stoma surgery and found that this intervention resulted in reduced length of stay (8 days vs. 10 days), reduced need for unplanned medical interventions after discharge, reduced the median time to attaining proficiency in managing the stoma (5.5 days vs. 9 days), and cost savings (94). Multiple observational and cross-sectional studies and one small randomized controlled trial supported the benefits of perioperative education for ostomy nurses (69, 93–97). Also, two randomized trials and several observational studies support the value of post-discharge stoma nurse care (98–102), and that such care can be achieved in a variety of ways. In the past, methods such as video CDs, presentations and PowerPoint slides have been used (103). With the proliferation of mobile communication devices, home follow-up care *via* a mobile app is emerging as superior. Patients intervening through mobile apps are not subject to geographic, time, cultural and social barriers and can receive more convenient and timely transitional care from the stoma nurse. Patients transmit pictures of their stoma and perioral skin *via* the mobile app to the stoma nurse, who assesses the patient’s stoma condition. The program allows patients to submit data frequently and provides more information than telephone or outpatient follow-up care (104). It can also allows one ostomy nurse to follow up with more patients in a short period of time. Timely advocacy and out-of-hospital follow-up are also essential for patients after TPS. A study by Zhang J et al. found that at 3 months postoperatively, patients who had received postoperative advocacy and follow-up were significantly better than the control group in terms of stoma adjustment, stoma self-healing, and satisfaction with care of stoma complications (99).

## 4 Problems facing transitory protective stoma

In the past, we were conservative about performing TPS, partly because of the series of stoma-related complications during the stoma period, and partly because of the postoperative psychological changes in the patient and the difficulties in caring for the stoma on their own after discharge.

The incidence of specific complications after stoma surgery is high, about 21%-70% (105), including skin infections, electrolyte imbalance, dehydration, renal failure, parastomal hernia, and stoma retraction or prolapse (39). Of these, dehydration is the most common stoma-related complication and a major factor in rehospitalization of patients (106–109). This is usually associated with decreased small bowel absorption due to the chemotherapy the patient received postoperatively. In patients receiving an ileostomy, the overall fluid balance is very fragile and even small changes caused by less intake or more output (e.g. use of diuretics, etc.) can trigger fluid imbalances that gradually lead to dehydration and the need for readmission and intravenous fluids. Dehydration is a preventable ileostomy-related complication. Optimizing stoma placement through enterostomy treatment counseling, patient education, and using the most terminal small bowel stoma to maximize absorption of intestinal fluids are the primary goals for preventing ileostomy complications. All ileostomy patients should have their stoma outflow and inflow measured daily to achieve a unique fluid balance for each patient (107).

Also, the closure of the stoma is an issue of concern. The evidence on the timing of stoma closure is limited. Surgeons believe that the stoma should close no earlier than 60 to 90 days after sphincter-sparing proctectomy. This timing represents that the patient has recovered from the trauma of the initial surgery, intra-abdominal adhesions are more easily managed, and anastomotic inflammation and edema have resolved (110, 111). Delayed closure of the stoma also exposes patients to a variety of stoma complications, including poor stoma position, dehydration, acute renal failure, the need for parenteral nutrition, peristomal dermatitis, parastomal hernias, prolapse, retraction, and stricture (105). In the field of gynecologic oncology, the optimal timing of reversal remains unclear. Almost all patients undergoing PDS receive adjuvant chemotherapy postoperatively, which can cause the stoma return time to be prolonged accordingly. Therefore, more evidence-based medical evidence is needed to support the time to stoma return in patients with TPS. Most studies currently document a mean stoma reversal time of 6 months (35, 37, 62). The optimal time to stoma reversion is recommended to be 3-6 months (112). Delay in return time beyond 6 months will increase the length of hospital stay and complication rate (113).

Return of stoma is associated with a range of potential surgical complications. Shama et al. drew extensive data from a representative national database and found a major complication rate of 9.3% and a mortality rate of 0.6% for return of the stoma. Patient functional status, ASA classification, and organ dysfunction were independent predictors of complications after ileostomy closure (114). The main causes of morbidity were wound infection, bowel obstruction, incisional hernia and anastomotic leak (115). The choice of anastomotic approach will directly influence the occurrence of postoperative complications. There have been four randomized controlled trials comparing mechanical and manual suture techniques. Overall, the results were similar, with a higher risk of postoperative obstruction and longer operative times with manual sutures (116–119). Surgical site infection is also a common complication after stoma reduction, reported to occur in 2%-41% of patients (120, 121). Wound infection after stoma closure can have serious consequences such as wound dehiscence, incisional hernia, prolonged hospital stay and increased hospital costs. Many surgeons search for suitable sutures to reduce the incidence of surgical site infections. Banerjee first described the technique of subcutaneous suturing of the purse-string to reduce the size of the wound. This is a similar closure to that of the severed end after appendectomy. After placing the reanastomosed bowel back into the abdominal cavity, the rectus is repaired with interrupted sutures. The skin defect is then apposed with subcuticular sutures and purse-string drawn together, leaving a 5-mm gap. Any residual hematoma or exudate can be drained through the gap (122). This type of suture was shown to be superior in subsequent studies. In Milanci’s study, no incisional infection was observed with the purse-string suture method, whereas a 40% incisional infection rate was observed with the conventional linear suture method (P=0.002). Moreover, the long-term follow-up results showed higher patient satisfaction and better postoperative healing and cosmetic outcomes with the purse-string suture (121). Similar conclusions were reached by Daniel et al. 36.67% of patients had postoperative infection with the conventional linear suture method, whereas none were diagnosed in the purse-string group. Also, the healing time was 2.1 week faster in the purse-string suture group than the linear suture group (P < 0.0001) (115). This shows that a more optimal suture protocol results in a more aesthetically pleasing healing wound and a lower chance of infection for the patient.

According to studies, 75.8%-92% of patients with enterostomies after OC surgery undergo a closure program (37, 57, 66, 123), but a proportion of patients still convert to permanent enterostomy due to many reasons such as long-term antiangiogenetic maintenance treatment etc. The main reason for failure to return is recurrence or progression of the disease. For patients with tumor recurrence, the closure plan cannot be implemented, because it may interrupt the continuous treatment of the tumor. Therefore, CT imaging of the abdomen and serum tumor markers should be performed to rule out recurrence or progression of the disease prior to the implementation of the return program (57). The risk of postoperative intestinal obstruction is greatly increased when a closure is performed in patients with progressive ovarian cancer. In patients receiving neoadjuvant chemotherapy, although it is widely accepted that neoadjuvant chemotherapy increases the likelihood of R_0_ resection, it is also an important risk factor for anastomotic healing, increasing the risk of anastomosis-related complications, and prolonging the time to stoma closure. While it reduces the probability of bowel resection in interval cytoreductive surgery (IDS), it does not reduce the incidence of enterostomy-related complications (124). In patients receiving cytoreductive surgery with heated intraperitoneal chemotherapy (HIPEC), the possibility of temporary stoma becoming permanent is greatly increased. In the study of Andrea et al., 78% of patients with stoma were converted to permanent stoma. In addition, stoma reversal in this group of patients is associated with significant major morbidity and mortality. This may be due to reduced physiologic reserve due to severe advanced malignancy pretreatment with previous surgery and multiple systemic chemotherapy. Therefore, patients with HIPEC with high disease burden should be carefully considered for stoma application and have a low probability of successful stoma reversal. Different stoma modalities and the patient’s own condition also influence the stoma return. In the study by M. F. Sier et al, independent predictors of non-reversal were terminal ileostomy, preoperative radiotherapy, body mass index and advanced age. Patients with loop ileostomy were 4.3 times more likely to have reversal than patients with terminal ileostomy. Also, the odds of reversal decreased by 3% per year with increasing age. The odds of reversal increased by 7% for each percentage point increase in body mass index (125). In addition, the lack of specialized treatment pathways for patients with TPS (37), cultural choices, and socioeconomic factors may contribute to non-reversal. The return of enterostomy is also often affected by chronic diseases such as diabetes (126). In the colorectal literature, advanced age and comorbidities are the most important risk factors for anastomotic non-reversal (127–129).

In addition to the impact of the surgery itself on the patient, the psychological impact of the stoma on the patient cannot be ignored. Research on the psychosocial problems of patients with stomas began about 50 years ago. For patients who eventually develop a stoma, they must initially deal with severe surgery, loss of important physical functions, image distortion, and significant and important changes in physical functioning and personal care (130–132). During hospitalization, the main stressors that affect patients’ emotions come from difficulty accepting a cancer diagnosis, adjusting to the presence of a stoma, dealing with various related emotions (e.g., shock, disgust, sadness, fear), learning practical skills for stoma self-care, and planning to return to normal activities. Slightly different stressors occurred after discharge, focusing on challenges with self-care and lifestyle adaptations. All of these changes affect aspects of the psychosocial domain of the patient’s life, sexual health, body image, or cultural and religious beliefs (131, 133–137). These physical and psychological stressors play a very important role in the adaptation and prognosis of the disease, as well as in the patient’s perceived quality of life (130, 138). They had a lower overall quality of life, poorer body image, reduced social activity, and significantly higher rates of depression and anxiety than patients without a stoma. Even a few months after discharge, patients had difficulty adjusting to changes in body image. Hortense et al. conducted a cross-sectional correlation study in Portugal to investigate the effects of colorectal cancer on the quality of life of 153 patients 6-8 months after surgery. The researchers found that patients with anastomosis had significantly lower levels of functioning and quality of life in terms of body image and sexual satisfaction, and significantly higher levels of depression compared to patients without anastomosis (139). Therefore, medical treatment of patients with stomas should be accompanied by positive guidance and education about their psychosocial needs, which can have a positive impact on quality of life and prognosis, and can reduce the length of hospitalization, frequency of postoperative complications and re-hospitalization (140).

## 5 Discussion

There is a paucity of gynecologic oncology literature on TPS, and criteria for the use of TPS in the gynecologic oncology research field still rely heavily on subjective operator judgment and lack standardized surgical criteria. Our assumptions and management decisions are largely extrapolated from the colorectal cancer literature. In patients with known high-risk factors, the use of TPS may reduce the severity of postoperative intestinal complications, give patients more opportunities for conservative treatment, and improve their prognosis. However, the complications associated with TPS and the troubles associated with the stoma reversal should not be ignored. At the same time, the use of TPS should be carefully selected considering the complications associated with TPS and the impact on patients’ quality of life and psychosocial well-being. There is no strong evidence to support the routine use of TPS in patients with OC combined with bowel surgery. In the future, more evidence from large prospective trials is needed to support the use of TPS and to clarify the indications for performing TPS surgery. Selection of appropriate patients based on indications is essential and requires a customized postoperative care plan specifically for the stoma population.

## Author contributions

LO put forward the proposition. JH drafted the manuscript. JL, BF, LY collected literatures. LO revised the final manuscript. All the authors contributed to this article and approved the submitted version.
